# Crystal structure, synthesis and thermal properties of tetra­kis­(4-benzoyl­pyridine-κ*N*)bis­(iso­thio­cyanato-κ*N*)iron(II)

**DOI:** 10.1107/S2056989019007679

**Published:** 2019-05-31

**Authors:** Carsten Wellm, Christian Näther

**Affiliations:** a Institut für Anorganische Chemie, Universität Kiel, Max-Eyth. Str. 2, 241128 Kiel, Germany

**Keywords:** crystal structure, iron(II) thio­cyanate, discrete complex, hydrogen bonding

## Abstract

The asymmetric unit of the title compound, [Fe(NCS)_2_(C_12_H_9_NO)_4_], consists of an Fe^II^ ion that is located on a centre of inversion, as well as two 4-benzoyl­pyridine ligands and one thio­cyanate anion in general positions. The Fe^II^ ions are coordinated by two N-terminal-bonded thio­cyanate anions and four 4-benzoyl­pyridine ligands into discrete complexes with a slightly distorted octa­hedral geometry. These complexes are further linked by weak C—H⋯O hydrogen bonds into chains running along the *c*-axis direction. Upon heating, this complex loses half of the 4-benzoyl­pyridine ligands and transforms into a compound with the composition Fe(NCS)_2_(4-benzoyl­pyridine)_2_, that might be isotypic to the corresponding Mn^II^ compound and for which the structure is unknown.

## Abstract 2

In the crystal structure of the title compound, the Fe^II^ ions are ocathedrally coordinated, forming discrete complexes that are linked into chains by inter­molecular C—H⋯O inter­actions.

## Chemical context   

Coordination compounds based on thio- or seleno­cyanate anions have attracted much inter­est in recent years because of their luminescence behavior and their versatile magnetic properties (Mekuimemba *et al.*, 2018 ▸; Palion-Gazda *et al.*, 2015 ▸, 2017 ▸; Mautner *et al.*, 2016*a* ▸,*b* ▸; Näther *et al.*, 2013 ▸). For the latter, compounds are of special inter­est in which paramagnetic transition-metal cations are linked by the anionic ligands into 1D or 2D coordination polymers. Some of them show single-chain-magnet behavior (Wöhlert *et al.*, 2013 ▸; 2014*a* ▸; Mautner *et al.*, 2018 ▸), others are ferromagnets (Suckert *et al.*, 2016 ▸) and in a few cases the critical temperature can be tuned by mixed-crystal formation (Neumann *et al.*, 2018*a* ▸, 2019 ▸; Wellm *et al.*, 2018 ▸).

However, in most cases compounds are obtained from solution in which the anionic ligands are only terminally N-bonded, which frequently leads to the formation of discrete complexes (Mautner *et al.*, 2015 ▸, 2017 ▸). These compounds can be transformed into coordination polymers by thermal decomposition, in which some of the co-ligands are irreversibly removed (Näther *et al.*, 2013 ▸), leading to the formation of polymorphic or isomeric modifications (Wöhlert *et al.*, 2014*b* ▸). In several cases Mn^II^, Fe^II^, Co^II^, Ni^II^ and Cd^II^ compounds behave similarly but in others, different modifications are obtained depending on the actual metal cation.

This is the case *e.g.* for thio­cyanate complexes with 4-benzoyl­pyridine as co-ligand. The discrete complexes with the composition *M*(NCS)_2_(4-benzoyl­pyridine)_4_ (*M* = Co and Ni) transform into isotypic chain compounds with the composition [*M*(NCS)_2_(4-benzoyl­pyridine)_2_]_*n*_, whereas both the Mn and Cd compounds each form a different crystalline phase (Neumann *et al.*, 2018*b* ▸; Wellm & Näther, 2018 ▸). Therefore, we became inter­ested in the corresponding complex with Fe^II^ to check if this compound could also transform into a 4-benzoyl­pyridine-deficient phase and if this phase would be isotypic to that with Mn^II^, Co^II^ or Cd^II^. The synthesis of the title compound can easily be achieved by the reaction of Fe(Cl)_2_·4H_2_O and K(SCN)_2_ with 4-benzoylpyridine, leading to the formation of phase pure samples (see Figure S1 in the supporting information). Upon heating, two mass losses are observed, of which the first one is in agreement with that expected for the removal of half of the 4-benzoyl­pyridine co-ligands (Figure S2). If the residue formed after the first thermogravimetric step is investigated by XRPD, it is obvious that a crystalline phase is formed (Figure S3) that is not isotypic to [*M*(NCS)_2_(4-benzoyl­pyridine)_2_]_*n*_ (*M* = Co, Cd) but very similar to that of Mn(NCS)_2_(4-benzoyl­pyridine)_2_ (Rams *et al.*, 2017 ▸; Neumann *et al.*, 2018**b* ▸;* Wellm & Näther, 2018 ▸). Additionally, IR spectra show that the residue exhibits bridging μ-1,3-coordinating thio­cyanate anions, in contrast to the terminal thio­cyanate anions of the title compound (Figure S4). Unfortunately, as is the case for the Mn^II^ compound, the powder pattern cannot be indexed and no single crystals can be obtained. Therefore, the structure of this compound is still unknown.
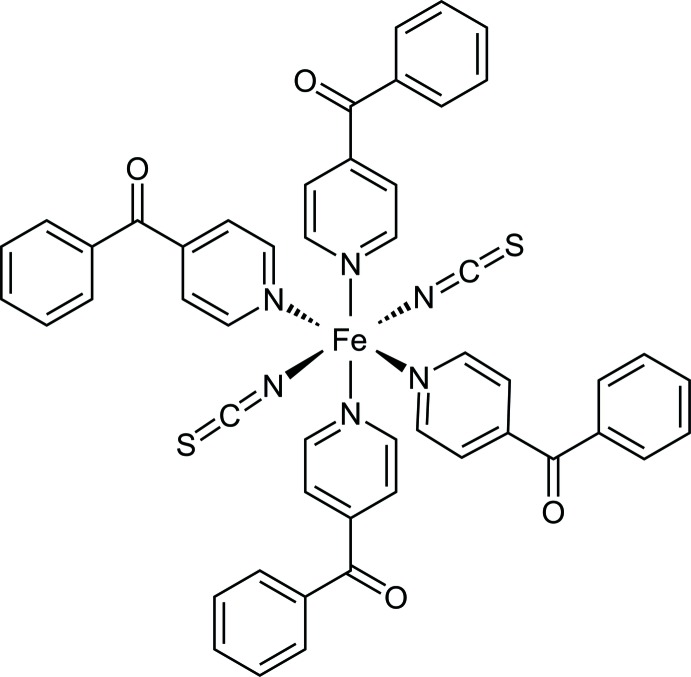


## Structural commentary   

**Figure 1:**
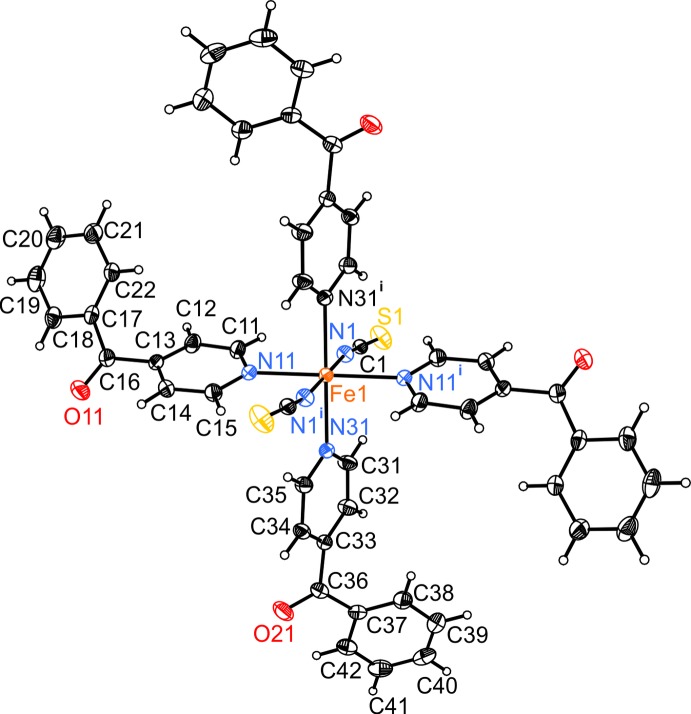
View of a discrete complex with the atom labeling and displacement ellipsoids drawn at the 50% probability level. Symmetry code: (i) −*x*, −*y* + 1, −*z* + 1.

The crystal structure of the title compound (Fig. 1 ▸) is isotypic to the corresponding Co^II^, Ni^II^, Mn^II^, Zn^II^ and Cd^II^ compounds (Drew *et al.*, 1985 ▸; Soliman *et al.*, 2014 ▸; Wellm & Näther, 2018 ▸; Neumann *et al.*, 2018*b* ▸). The asymmetric unit consists of one N-bonded terminal thio­cyanate anion and two crystallographically independent 4-benzoyl­pyridine ligands in general positions, as well as of one Fe^II^ cation located on a centre of inversion (Fig. 1 ▸). The Fe^II^ ions are sixfold coordin­ated by the pyridine N-atoms of the four neutral 4-benzoyl­pyridine ligands and the N atoms of the two terminal thio­cyanate anions. The Fe—N bonds to the 4-benzoyl­pyridine coligands, ranging between 2.2576 (13) and 2.2597 (13) Å, are significantly longer than those to the anionic ligands of 2.0982 (14) Å (Table 1 ▸) and correspond to those observed in the isotypic compounds [*M*(NCS)_2_(C_12_H_9_NO)_4_] (*M* = Mn, Co, Ni, Zn, Cd; Wellm & Näther, 2018 ▸; Drew *et al.*, 1985 ▸; Soliman *et al.*, 2014 ▸; Neumann *et al.*, 2018*b* ▸). The N—*M*—N angles deviate from the ideal values, which shows that the octa­hedra are slightly distorted in agreement with the values for the angle variance (1.8) and the quadratic elongation (1.003) (Robinson *et al.*, 1971 ▸). Furthermore, the pyridine and phenyl rings of the 4-benzoyl­pyridine ligands are not co-planar to the carbonyl plane. The dihedral angle between the pyrdine ring (N11/C11–15) and the carbonyl plane (C13/C16/C17/O11) amounts to 35.24 (10)°, while the one between the carbonyl plane (C13/C16/C17/O11) and the phenyl ring (C17–C22) is 24.23 (8)°. The corresponding values for the second 4-benzoyl­pyrdine ligand are 35.69 (9)° between the pyridine ring (N31/C31–C35) and the carbonyl plane (C33/C36/C37/O21) and 23.79 (9)° between the carbonyl plane (C33/C36/C37/O21) and the phenyl ring (C37–C42). Additionally, there are weak intra­molecular C—H⋯N inter­actions between the thio­cyanate atoms N1 and N1^i^ and aromatic hydrogen atoms H11, H31, H15 and H35 that might contribute to the stabilization of the complexes (Table 2 ▸).

## Supra­molecular features   

**Figure 2:**
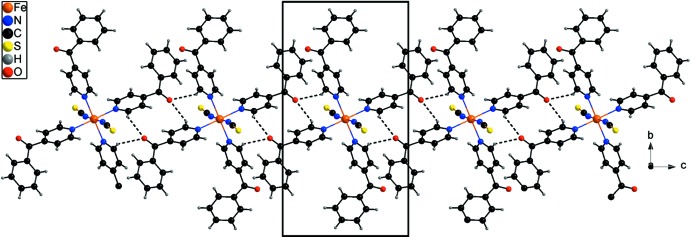
Crystal packing of the title compound viewed along the crystallographic *a* axis with inter­molecular C—H⋯O hydrogen bonds (Table 2 ▸) shown as dashed lines.

**Figure 3:**
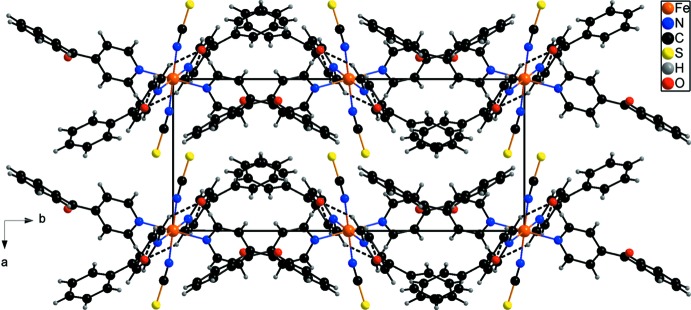
Crystal packing of the title compound viewed along the crystallographic *c* axis with inter­molecular C—H⋯O hydrogen bonds (Table 2 ▸) shown as dashed lines.

The discrete complexes are connected by relatively weak C—H⋯O hydrogen bonds between the C—H hydrogen atoms and the atom O21(−*x*, 1 − *y*, 2 − *z*) of a symmetry-related 4-benzoyl­pyridine ligand, forming 12-membered rings that are located on centres of inversion (Fig. 2 ▸ and Table 2 ▸). Atom O21 acts as acceptor for two hydrogen bonds from C15—H15 and C35—H35; thus each complex is connected by four hydrogen bonds to two additional symmetry-equivalent complexes, leading to the formation of chains that extend along the *c*-axis direction (Figs. 2 ▸ and 3 ▸ and Table 2 ▸). There are no further directed inter­actions observed between the chains (Fig. 3 ▸).

## Database survey   

There are several crystal structures reported in the Cambridge Structure Database (Version 5.40, last update February 2018; Groom *et al.*, 2016 ▸) that consist of transition-metal cations, thio­cyanate anions and 4-benzoyl­pyrine. In most of these compounds, the metal cations are octa­hedrally coordinated. Three of them are coordination polymers in which the cations are connected by pairs of μ-1,3-coordinating thio­cyanate anions, with the 4-benzoyl­pyridine ligands being perpendicular to the elongation axis of the chain (Neumann *et al.*, 2018*b* ▸; Rams *et al.*, 2017 ▸; Jochim *et al.*, 2018 ▸). The other octa­hedral compounds are either discrete complexes with only 4-benzoyl­pyridine as neutral co-ligand, isotypic to the title compound and of the general composition *M*(NCS)_2_(4-benzoyl­pyridine)_4_ (*M* = Co^II^, Ni^II^, Mn^II^, Zn^II^ and Cd^II^; Drew *et al.*, 1985 ▸; Soliman *et al.*, 2014 ▸; Wellm & Näther, 2018 ▸; Neumann *et al.*, 2018*b* ▸), or solvates that are built up of two terminally N-bonded thio­cyanates, two 4-benzoyl­pyridine ligands and aceto­nitrile (Suckert *et al.*, 2017*b* ▸) or methanol as solvent (Suckert *et al.*, 2017*a* ▸; Wellm & Näther, 2019 ▸). Additionally, there is a quadratic planar Cu^II^ complex (Bai *et al.*, 2011 ▸) and a tetra­hedral Zn^II^ complex (Neumann *et al.*, 2018*b* ▸) in which the metal cation is coordinated by two terminally N-bonded thio­cyanates and two 4-benzoyl­pyridine ligands.

## Synthesis and crystallization   

Fe(Cl)_2_·4H_2_O and K(SCN)_2_ were purchased from Merck and 4-benzoyl­pyridine was purchased from Alfa Aesar.

**Synthesis:**

Crystals of the title compound suitable for single crystal X-ray diffraction were obtained within three days by the reaction of 59.6 mg Fe(Cl)_2_·4H_2_O (0.3 mmol) and 58.3 mg (0.6 mmol) K(SCN)_2_ with 27.5 mg 4-benzoyl­pyridine (0.15 mmol) in ethanol (1.5 mL), followed by slow evaporation of the solvent.

**Experimental details:**

Differential thermal analysis-thermogravimetric (DTA-TG) measurements were performed in a dynamic nitro­gen atmosphere in Al_2_O_3_ crucibles using an STA PT1600 thermobalance from Linseis. The XRPD measurements were performed by using a Stoe transmission powder diffraction system (STADI P) with Cu *K*α radiation that was equipped with a linear, position-sensitive MYTHEN detector from Stoe & Cie. The IR data were measured using a Bruker Alpha-P ATR-IR spectrometer.

## Refinement   

Crystal data, data collection and structure refinement details are summarized in Table 3 ▸. Hydrogen atoms were positioned with idealized geometry (C—H = 0.95 Å) and were refined using a riding model with *U*_iso_(H) = 1.2*U*_eq_(C).

## Supplementary Material

Crystal structure: contains datablock(s) I. DOI: 10.1107/S2056989019007679/lh5906sup1.cif

Structure factors: contains datablock(s) I. DOI: 10.1107/S2056989019007679/lh5906Isup2.hkl

Figure S1. Experimental (top) and calculated X-ray powder pattern of the title compound (Cu-Kalpha radiation). DOI: 10.1107/S2056989019007679/lh5906sup3.tif

Figure S2. DTG, TG and DTA curve of the title compound measured with 1 C/min in a nitrogen atmosphere. The mass loss calculated for the removal of one 4-benzoylpyridine ligand corresponds to 19.9%. DOI: 10.1107/S2056989019007679/lh5906sup4.tif

Figure S3. X-ray powder patterns of the residue obtained after the first mass loss of the title compound at 1 C (A), of the residue obtained after the first mass loss of [Mn(SCN)2(4-benzoylpyridine)4] at 1 C (B) and the calculated powder patterns for [Co(NCS)2(4-benzoylpyridine)2] (C) and [Cd(NCS)2(4-benzoylpyridine)2] (D) (Cu-Kalpha radiation). DOI: 10.1107/S2056989019007679/lh5906sup5.tif

Figure S4. IR spectra of the residue obtained after the first mass loss of the title compound (top) in comparism to the title compound (bottom). DOI: 10.1107/S2056989019007679/lh5906sup6.tif

CCDC reference: 1918968

Additional supporting information:  crystallographic information; 3D view; checkCIF report

## Figures and Tables

**Table 1:** Selected geometric parameters (Å, °)

Fe1—N1	2.0982 (14)	Fe1—N31	2.2597 (13)
Fe1—N11	2.2576 (13)		
			
N1^i^—Fe1—N11	88.79 (5)	N1—Fe1—N31	89.79 (5)
N1—Fe1—N11	91.21 (5)	N11—Fe1—N31	88.15 (5)
N1^i^—Fe1—N31	90.21 (5)	N11^i^—Fe1—N31	91.85 (5)

Symmetry code: (i) 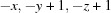.

**Table 2:** Hydrogen-bond geometry (Å, °)

*D*—H⋯*A*	*D*—H	H⋯*A*	*D*⋯*A*	*D*—H⋯*A*
C11—H11⋯N1	0.95	2.51	3.136 (2)	123
C15—H15⋯N1^i^	0.95	2.57	3.134 (2)	118
C15—H15⋯O21^ii^	0.95	2.57	3.293 (2)	133
C31—H31⋯N1	0.95	2.60	3.168 (2)	119
C35—H35⋯N1^i^	0.95	2.52	3.125 (2)	121
C35—H35⋯O21^ii^	0.95	2.61	3.309 (2)	131

Symmetry codes: (i) 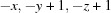; (ii) 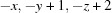.

**Table 3:** Experimental details

Crystal data	
Chemical formula	[Fe(NCS)_2_(C_12_H_9_NO)_4_]
*M*_r_	904.82
Crystal system, space group	Monoclinic, *P*2_1_/*c*
Temperature (K)	200
*a*, *b*, *c* (Å)	9.0610 (6), 20.9844 (11), 11.2527 (9)
β (°)	90.526 (9)
*V* (Å^3^)	2139.5 (2)
*Z*	2
Radiation type	Mo *K*α
μ (mm^−1^)	0.51
Crystal size (mm)	0.16 × 0.04 × 0.03

Data collection	
Diffractometer	STOE IPDS1
Absorption correction	Numerical (*X-SHAPE* and *X-RED32*; Stoe, 2008 ▸)
*T*_min_, *T*_max_	0.817, 0.965
No. of measured, independent and observed [*I* > 2σ(*I*)] reflections	25216, 4907, 4090
*R*_int_	0.060
(sin θ/λ)_max_ (Å^−1^)	0.650

Refinement	
*R*[*F* ^2^ > 2σ(*F* ^2^)], *wR*(*F* ^2^), *S*	0.044, 0.113, 1.04
No. of reflections	4907
No. of parameters	287
H-atom treatment	H-atom parameters constrained
Δρ_max_, Δρ_min_ (e Å^−3^)	0.50, −0.51

Computer programs: *X-AREA* (Stoe, 2008 ▸), *XP* in *SHELXTL* and *SHELXS97* (Sheldrick, 2008 ▸), *SHELXL2014* (Sheldrick, 2015 ▸), *DIAMOND* (Brandenburg, 1999 ▸) and *publCIF* (Westrip, 2010 ▸).

## supplementary crystallographic information

### Crystal data

**Table:**

[Fe(NCS)_2_(C_12_H_9_NO)_4_]	*F*(000) = 936
*M**_r_* = 904.82	*D*_x_ = 1.405 Mg m^−^^3^
Monoclinic, *P*2_1_/*c*	Mo *K*α radiation, λ = 0.71073 Å
*a* = 9.0610 (6) Å	Cell parameters from 25216 reflections
*b* = 20.9844 (11) Å	θ = 2.5–27.5°
*c* = 11.2527 (9) Å	µ = 0.51 mm^−^^1^
β = 90.526 (9)°	*T* = 200 K
*V* = 2139.5 (2) Å^3^	Needle, light yellow
*Z* = 2	0.16 × 0.04 × 0.03 mm

### Data collection

**Table:**

STOE IPDS-1 diffractometer	4090 reflections with *I* > 2σ(*I*)
φ scans	*R*_int_ = 0.060
Absorption correction: numerical (X-SHAPE and X-RED32; Stoe, 2008)	θ_max_ = 27.5°, θ_min_ = 2.5°
*T*_min_ = 0.817, *T*_max_ = 0.965	*h* = −11→11
25216 measured reflections	*k* = −27→27
4907 independent reflections	*l* = −14→14

### Refinement

**Table:**

Refinement on *F*^2^	Hydrogen site location: inferred from neighbouring sites
Least-squares matrix: full	H-atom parameters constrained
*R*[*F*^2^ > 2σ(*F*^2^)] = 0.044	*w* = 1/[σ^2^(*F*_o_^2^) + (0.0769*P*)^2^ + 0.3118*P*] where *P* = (*F*_o_^2^ + 2*F*_c_^2^)/3
*wR*(*F*^2^) = 0.113	(Δ/σ)_max_ < 0.001
*S* = 1.04	Δρ_max_ = 0.50 e Å^−^^3^
4907 reflections	Δρ_min_ = −0.51 e Å^−^^3^
287 parameters	Extinction correction: SHELXL, Fc^*^=kFc[1+0.001xFc^2^λ^3^/sin(2θ)]^-1/4^
0 restraints	Extinction coefficient: 0.026 (2)

### Special details

**Table:**

Geometry. All esds (except the esd in the dihedral angle between two l.s. planes) are estimated using the full covariance matrix. The cell esds are taken into account individually in the estimation of esds in distances, angles and torsion angles; correlations between esds in cell parameters are only used when they are defined by crystal symmetry. An approximate (isotropic) treatment of cell esds is used for estimating esds involving l.s. planes.

### Fractional atomic coordinates and isotropic or equivalent isotropic displacement parameters (Å^2^)

**Table:**

	*x*	*y*	*z*	*U*_iso_*/*U*_eq_	
Fe1	0.0000	0.5000	0.5000	0.01655 (12)	
N1	0.21274 (16)	0.51129 (6)	0.43099 (13)	0.0221 (3)	
C1	0.32862 (18)	0.52598 (7)	0.39685 (14)	0.0204 (3)	
S1	0.48986 (5)	0.54766 (3)	0.34977 (5)	0.03812 (15)	
N11	0.05996 (15)	0.40356 (6)	0.57514 (12)	0.0204 (3)	
C11	0.18068 (19)	0.37360 (7)	0.53597 (16)	0.0248 (3)	
H11	0.2429	0.3953	0.4819	0.030*	
C12	0.2190 (2)	0.31228 (8)	0.57078 (17)	0.0270 (4)	
H12	0.3043	0.2924	0.5393	0.032*	
C13	0.13182 (19)	0.28026 (7)	0.65168 (15)	0.0224 (3)	
C14	0.0108 (2)	0.31223 (8)	0.69739 (15)	0.0255 (3)	
H14	−0.0487	0.2927	0.7562	0.031*	
C15	−0.02230 (19)	0.37287 (8)	0.65646 (15)	0.0246 (3)	
H15	−0.1068	0.3938	0.6871	0.030*	
C16	0.1689 (2)	0.21450 (8)	0.69528 (16)	0.0288 (4)	
C17	0.2345 (2)	0.16681 (7)	0.61300 (16)	0.0252 (4)	
C18	0.3114 (2)	0.11520 (9)	0.66334 (19)	0.0319 (4)	
H18	0.3276	0.1133	0.7468	0.038*	
C19	0.3635 (2)	0.06699 (9)	0.5907 (2)	0.0393 (5)	
H19	0.4160	0.0322	0.6246	0.047*	
C20	0.3395 (2)	0.06935 (9)	0.4693 (2)	0.0400 (5)	
H20	0.3751	0.0360	0.4202	0.048*	
C21	0.2638 (2)	0.11996 (9)	0.41879 (19)	0.0367 (4)	
H21	0.2475	0.1212	0.3353	0.044*	
C22	0.2112 (2)	0.16913 (8)	0.49028 (16)	0.0286 (4)	
H22	0.1599	0.2041	0.4556	0.034*	
O11	0.1429 (2)	0.20092 (7)	0.79860 (13)	0.0537 (5)	
N31	0.07705 (15)	0.54355 (6)	0.67343 (12)	0.0206 (3)	
C31	0.2025 (2)	0.57704 (8)	0.68418 (15)	0.0274 (4)	
H31	0.2607	0.5831	0.6153	0.033*	
C32	0.2519 (2)	0.60332 (9)	0.79080 (16)	0.0277 (4)	
H32	0.3403	0.6276	0.7937	0.033*	
C33	0.17005 (18)	0.59348 (7)	0.89320 (14)	0.0217 (3)	
C34	0.04162 (19)	0.55810 (8)	0.88302 (15)	0.0239 (3)	
H34	−0.0168	0.5500	0.9511	0.029*	
C35	−0.00132 (19)	0.53446 (8)	0.77268 (15)	0.0232 (3)	
H35	−0.0904	0.5107	0.7672	0.028*	
C36	0.21784 (19)	0.61552 (8)	1.01527 (15)	0.0252 (3)	
C37	0.29466 (19)	0.67775 (8)	1.03193 (15)	0.0242 (3)	
C38	0.2782 (2)	0.72793 (9)	0.95250 (17)	0.0335 (4)	
H38	0.2207	0.7224	0.8822	0.040*	
C39	0.3458 (3)	0.78642 (9)	0.9755 (2)	0.0383 (5)	
H39	0.3335	0.8208	0.9214	0.046*	
C40	0.4306 (2)	0.79423 (9)	1.07696 (19)	0.0363 (4)	
H40	0.4772	0.8340	1.0923	0.044*	
C41	0.4480 (2)	0.74438 (10)	1.15663 (18)	0.0372 (4)	
H41	0.5067	0.7500	1.2262	0.045*	
C42	0.3800 (2)	0.68632 (9)	1.13492 (16)	0.0309 (4)	
H42	0.3913	0.6523	1.1900	0.037*	
O21	0.19095 (18)	0.58129 (7)	1.10006 (12)	0.0400 (3)	

### Atomic displacement parameters (Å^2^)

**Table:**

	*U*^11^	*U*^22^	*U*^33^	*U*^12^	*U*^13^	*U*^23^
Fe1	0.01555 (17)	0.01668 (16)	0.01743 (17)	−0.00085 (10)	0.00129 (12)	−0.00031 (11)
N1	0.0182 (7)	0.0252 (6)	0.0229 (7)	0.0000 (5)	0.0008 (5)	0.0007 (5)
C1	0.0222 (8)	0.0205 (7)	0.0183 (7)	0.0020 (6)	−0.0022 (6)	0.0004 (6)
S1	0.0200 (2)	0.0558 (3)	0.0387 (3)	−0.00491 (19)	0.00399 (19)	0.0148 (2)
N11	0.0235 (7)	0.0174 (6)	0.0201 (6)	0.0003 (5)	−0.0002 (5)	−0.0009 (5)
C11	0.0256 (8)	0.0194 (7)	0.0294 (9)	0.0008 (6)	0.0058 (7)	0.0008 (6)
C12	0.0267 (9)	0.0205 (7)	0.0340 (9)	0.0041 (6)	0.0060 (7)	0.0000 (7)
C13	0.0282 (8)	0.0174 (7)	0.0217 (8)	−0.0004 (6)	−0.0032 (6)	−0.0011 (6)
C14	0.0306 (9)	0.0236 (8)	0.0225 (8)	−0.0013 (6)	0.0032 (7)	0.0027 (6)
C15	0.0243 (8)	0.0245 (8)	0.0250 (8)	0.0024 (6)	0.0042 (6)	0.0013 (6)
C16	0.0381 (10)	0.0224 (8)	0.0257 (8)	0.0001 (7)	−0.0038 (7)	0.0026 (6)
C17	0.0260 (8)	0.0177 (7)	0.0318 (9)	−0.0011 (6)	−0.0020 (7)	0.0024 (6)
C18	0.0300 (9)	0.0259 (8)	0.0398 (11)	0.0019 (7)	−0.0070 (8)	0.0071 (7)
C19	0.0299 (10)	0.0269 (9)	0.0610 (14)	0.0106 (7)	0.0012 (9)	0.0075 (9)
C20	0.0395 (11)	0.0279 (9)	0.0527 (13)	0.0067 (8)	0.0126 (10)	−0.0018 (8)
C21	0.0459 (12)	0.0281 (9)	0.0362 (10)	0.0045 (8)	0.0086 (9)	−0.0008 (8)
C22	0.0358 (10)	0.0212 (7)	0.0290 (9)	0.0031 (6)	0.0008 (7)	0.0033 (6)
O11	0.1029 (15)	0.0328 (7)	0.0256 (7)	0.0158 (8)	0.0054 (8)	0.0069 (6)
N31	0.0232 (7)	0.0191 (6)	0.0194 (7)	−0.0018 (5)	0.0016 (5)	−0.0012 (5)
C31	0.0299 (9)	0.0330 (9)	0.0193 (8)	−0.0106 (7)	0.0052 (7)	−0.0038 (6)
C32	0.0270 (9)	0.0338 (9)	0.0224 (8)	−0.0118 (7)	0.0020 (7)	−0.0033 (7)
C33	0.0252 (8)	0.0209 (7)	0.0189 (7)	0.0012 (6)	−0.0006 (6)	−0.0003 (6)
C34	0.0261 (8)	0.0266 (7)	0.0189 (7)	−0.0017 (6)	0.0043 (6)	0.0006 (6)
C35	0.0221 (8)	0.0260 (8)	0.0215 (8)	−0.0044 (6)	0.0019 (6)	−0.0011 (6)
C36	0.0263 (9)	0.0303 (8)	0.0189 (8)	0.0010 (6)	0.0001 (6)	0.0003 (6)
C37	0.0240 (8)	0.0290 (8)	0.0194 (8)	0.0012 (6)	0.0010 (6)	−0.0042 (6)
C38	0.0422 (11)	0.0304 (9)	0.0278 (9)	−0.0002 (8)	−0.0076 (8)	−0.0024 (7)
C39	0.0482 (12)	0.0274 (9)	0.0394 (11)	−0.0012 (8)	0.0007 (9)	−0.0014 (8)
C40	0.0321 (10)	0.0359 (10)	0.0409 (11)	−0.0051 (8)	0.0077 (8)	−0.0161 (8)
C41	0.0305 (10)	0.0503 (11)	0.0309 (10)	−0.0052 (8)	−0.0041 (8)	−0.0124 (8)
C42	0.0315 (10)	0.0387 (10)	0.0224 (8)	0.0001 (7)	−0.0033 (7)	−0.0033 (7)
O21	0.0545 (9)	0.0444 (8)	0.0210 (6)	−0.0136 (7)	−0.0015 (6)	0.0055 (6)

### Geometric parameters (Å, º)

**Table:**

Fe1—N1^i^	2.0982 (14)	C20—H20	0.9500
Fe1—N1	2.0982 (14)	C21—C22	1.395 (3)
Fe1—N11	2.2576 (13)	C21—H21	0.9500
Fe1—N11^i^	2.2576 (13)	C22—H22	0.9500
Fe1—N31	2.2597 (13)	N31—C31	1.341 (2)
Fe1—N31^i^	2.2597 (13)	N31—C35	1.343 (2)
N1—C1	1.163 (2)	C31—C32	1.391 (2)
C1—S1	1.6237 (17)	C31—H31	0.9500
N11—C11	1.340 (2)	C32—C33	1.392 (2)
N11—C15	1.349 (2)	C32—H32	0.9500
C11—C12	1.388 (2)	C33—C34	1.384 (2)
C11—H11	0.9500	C33—C36	1.509 (2)
C12—C13	1.384 (2)	C34—C35	1.389 (2)
C12—H12	0.9500	C34—H34	0.9500
C13—C14	1.388 (2)	C35—H35	0.9500
C13—C16	1.502 (2)	C36—O21	1.221 (2)
C14—C15	1.385 (2)	C36—C37	1.491 (2)
C14—H14	0.9500	C37—C38	1.388 (3)
C15—H15	0.9500	C37—C42	1.399 (2)
C16—O11	1.222 (2)	C38—C39	1.395 (3)
C16—C17	1.491 (2)	C38—H38	0.9500
C17—C22	1.396 (3)	C39—C40	1.380 (3)
C17—C18	1.404 (2)	C39—H39	0.9500
C18—C19	1.386 (3)	C40—C41	1.386 (3)
C18—H18	0.9500	C40—H40	0.9500
C19—C20	1.383 (3)	C41—C42	1.386 (3)
C19—H19	0.9500	C41—H41	0.9500
C20—C21	1.383 (3)	C42—H42	0.9500
			
N1^i^—Fe1—N1	180.0	C19—C20—H20	119.8
N1^i^—Fe1—N11	88.79 (5)	C21—C20—H20	119.8
N1—Fe1—N11	91.21 (5)	C20—C21—C22	120.1 (2)
N1^i^—Fe1—N11^i^	91.21 (5)	C20—C21—H21	119.9
N1—Fe1—N11^i^	88.79 (5)	C22—C21—H21	119.9
N11—Fe1—N11^i^	180.0	C21—C22—C17	119.71 (17)
N1^i^—Fe1—N31	90.21 (5)	C21—C22—H22	120.1
N1—Fe1—N31	89.79 (5)	C17—C22—H22	120.1
N11—Fe1—N31	88.15 (5)	C31—N31—C35	116.99 (14)
N11^i^—Fe1—N31	91.85 (5)	C31—N31—Fe1	123.01 (11)
N1^i^—Fe1—N31^i^	89.79 (5)	C35—N31—Fe1	119.96 (11)
N1—Fe1—N31^i^	90.21 (5)	N31—C31—C32	123.48 (16)
N11—Fe1—N31^i^	91.85 (5)	N31—C31—H31	118.3
N11^i^—Fe1—N31^i^	88.15 (5)	C32—C31—H31	118.3
N31—Fe1—N31^i^	180.00 (7)	C31—C32—C33	119.05 (16)
C1—N1—Fe1	170.93 (13)	C31—C32—H32	120.5
N1—C1—S1	179.08 (15)	C33—C32—H32	120.5
C11—N11—C15	117.19 (14)	C34—C33—C32	117.73 (15)
C11—N11—Fe1	119.46 (11)	C34—C33—C36	118.27 (15)
C15—N11—Fe1	123.32 (11)	C32—C33—C36	123.88 (15)
N11—C11—C12	123.01 (16)	C33—C34—C35	119.59 (15)
N11—C11—H11	118.5	C33—C34—H34	120.2
C12—C11—H11	118.5	C35—C34—H34	120.2
C13—C12—C11	119.53 (16)	N31—C35—C34	123.15 (15)
C13—C12—H12	120.2	N31—C35—H35	118.4
C11—C12—H12	120.2	C34—C35—H35	118.4
C12—C13—C14	117.79 (15)	O21—C36—C37	120.89 (16)
C12—C13—C16	122.26 (16)	O21—C36—C33	118.27 (16)
C14—C13—C16	119.87 (16)	C37—C36—C33	120.84 (14)
C15—C14—C13	119.35 (16)	C38—C37—C42	119.42 (17)
C15—C14—H14	120.3	C38—C37—C36	122.42 (16)
C13—C14—H14	120.3	C42—C37—C36	118.07 (16)
N11—C15—C14	123.00 (16)	C37—C38—C39	120.24 (18)
N11—C15—H15	118.5	C37—C38—H38	119.9
C14—C15—H15	118.5	C39—C38—H38	119.9
O11—C16—C17	121.05 (16)	C40—C39—C38	119.87 (19)
O11—C16—C13	118.74 (17)	C40—C39—H39	120.1
C17—C16—C13	120.21 (15)	C38—C39—H39	120.1
C22—C17—C18	119.72 (17)	C39—C40—C41	120.32 (18)
C22—C17—C16	122.25 (15)	C39—C40—H40	119.8
C18—C17—C16	117.80 (17)	C41—C40—H40	119.8
C19—C18—C17	119.70 (19)	C40—C41—C42	120.11 (18)
C19—C18—H18	120.1	C40—C41—H41	119.9
C17—C18—H18	120.1	C42—C41—H41	119.9
C20—C19—C18	120.35 (18)	C41—C42—C37	120.03 (18)
C20—C19—H19	119.8	C41—C42—H42	120.0
C18—C19—H19	119.8	C37—C42—H42	120.0
C19—C20—C21	120.41 (19)		

Symmetry code: (i) −*x*, −*y*+1, −*z*+1.

### Hydrogen-bond geometry (Å, º)

**Table:**

*D*—H···*A*	*D*—H	H···*A*	*D*···*A*	*D*—H···*A*
C11—H11···N1	0.95	2.51	3.136 (2)	123
C15—H15···N1^i^	0.95	2.57	3.134 (2)	118
C15—H15···O21^ii^	0.95	2.57	3.293 (2)	133
C31—H31···N1	0.95	2.60	3.168 (2)	119
C35—H35···N1^i^	0.95	2.52	3.125 (2)	121
C35—H35···O21^ii^	0.95	2.61	3.309 (2)	131

Symmetry codes: (i) −*x*, −*y*+1, −*z*+1; (ii) −*x*, −*y*+1, −*z*+2.
